# The Influence of Unstable Load and Traditional Free-Weight Back Squat Exercise on Subsequent Countermovement Jump Performance

**DOI:** 10.3390/jfmk8040167

**Published:** 2023-12-18

**Authors:** Renata Jirovska, Anthony D. Kay, Themistoklis Tsatalas, Alex J. Van Enis, Christos Kokkotis, Giannis Giakas, Minas A. Mina

**Affiliations:** 1 Department of Sport, Outdoor and Exercise Science, School of Human Sciences & Human Sciences Research Centre, University of Derby, Kedleston Road, Derby DE22 1GB, UK; renca274@gmail.com (R.J.); a.vanenis@derby.ac.uk (A.J.V.E.); 2 Sport, Exercise & Life Sciences, University of Northampton, Northampton NN1 5PH, UK; tony.kay@northampton.ac.uk; 3 Department of Physical Education and Sport Science, University of Thessaly, Karyes, 42100 Trikala, Greece; ttsatalas@uth.gr; 4 Department of Physical Education and Sport Science, Democritus University of Thrace, 69100 Komotini, Greece; ckokkoti@affil.duth.gr

**Keywords:** conditioning contractions, explosive strength, elastic bands, vertical jump, warm-up, post-activation performance enhancement (PAPE)

## Abstract

The purpose of the present study was to examine the effects of a back squat exercise with unstable load (UN) and traditional free-weight resistance (FWR) on subsequent countermovement jump (CMJ) performance. After familiarisation, thirteen physically active males with experience in resistance training visited the laboratory on two occasions during either experimental (UN) or control (FWR) conditions separated by at least 72 h. In both sessions, participants completed a task-specific warm-up routine followed by three maximum CMJs (pre-intervention; baseline) and a set of three repetitions of either UN or FWR back squat exercise at 85% 1-RM. During the UN condition, the unstable load was suspended from the bar with elastic bands and accounted for 15% of the total load. Post-intervention, three maximum CMJs were performed at 30 s, 4 min, 8 min and 12 min after the last repetition of the intervention. The highest CMJ for each participant was identified for each timepoint. No significant increases (*p* > 0.05) in jump height, peak concentric power, or peak rate of force development (RFD) were found after the FWR or UN conditions at any timepoint. The lack of improvements following both FWR and UN conditions may be a consequence of the low percentage of unstable load and the inclusion of a comprehensive task-specific warm-up. Further research is required to explore higher UN load percentages (>15%) and the chronic effects following the implementation of a resistance training programme.

## 1. Introduction

Warm-up protocols can precondition the neuromuscular system by manipulating different loading strategies to reduce the risk of injury and enhance performance in subsequent high-intensity activities [1,2,3]. Performing maximal or sub-maximal contractions can acutely increase force production and athletic performance as well as enhance mechanical power above previous voluntary performance, which is usually referred to as post-activation potentiation (PAP) although not synonymous with “classic” PAP (i.e., electrically elicited twitch contraction [4]). The term PAP and its associated mechanisms (including increased muscle temperature [5], myofilament calcium sensitivity [6], and neural drive [7]) have been misinterpreted in the literature and often used to describe an enhancement in voluntary muscle function instead of increases in electrically induced twitch force. However, acute enhancement in performance has been more recently reported as post-activation performance enhancement (PAPE) [8] following high-intensity voluntary muscular contractions and, importantly, can be incorporated in the design of warm-up strategies [9].

Whilst classical PAP is apparent for <3 min following the conditioning contraction [10], peak voluntary contraction (PAPE) occurs 6–10 min following the conditioning contraction [11]. Therefore, acute enhancements in voluntary performance are unlikely to be associated with classical PAP but rather the PAPE phenomenon. The mechanisms underpinning the PAPE phenomenon include (a) rapid increases in muscle temperature in response to a brief intense conditioning activity, which is associated with a greater rate of force development (RFD) and contraction velocity [12]; (b) a high-intensity stimulus (i.e., heavy-load exercise) increases H-reflex potentiation, the excitability of alpha motor neurons and the recruitment of higher-order motor units [13] to increase the efficiency of the neuromuscular system [14]; (c) increases in muscle blood flow and muscle fibre water content may also consequently increase Ca^2+^ sensitivity and thus enhance muscle force output and contraction velocity [15]; however, increases in motivation and acute improvements in motor control strategies cannot be discounted [9].

Warm-up is the process of physical preparation before sporting participation [16] and is considered to enhance subsequent performance [17]. Different limited warm-up strategies have been explored to acutely augment athletic performance ranging from no warm-up at all [4] to stretching, cycling, running, and sub-maximal repetitions of the task [3,18]. Jo et al. [3] found that recovery duration (5–20 min) failed to influence performance after a heavy-load back squat exercise with limited warm-up consisting of cycling for 10 min followed by a Wingate Test. Duthie et al. [18] implemented a standardised warm-up including cycling followed by static stretching and found a significant difference in power performance in jump squats using contrast training methods in athletes with higher strength levels compared to complex training methods. However, Hamada et al. [4] used no warm-up and found a greater potentiation response in Type I muscle fibres following a twitch maximum voluntary contraction. A “comprehensive task-specific” warm-up (including progressively intense task-specific conditioning contractions) has not been commonly used prior to a specific activity being tested [2]. Consequently, as warm-up strategies have been implemented to potentiate muscular force production to enhance subsequent performance following a conditioning activity, it is unclear whether any acute enhancements in performance are due to the warm-up or the conditioning activity itself [19].

The modalities necessary to elicit a PAPE effect remain relatively unexplored: particularly, varying repetitions and sets (volume), exercise intensity and rest periods [20]. Dynamic [21] and isometric voluntary contractions (MVCs) [22] have been used as conditioning contractions to elicit a PAPE response. The volume of conditioning contractions plays a key role in the onset and magnitude of PAPE for strength and conditioning practitioners on improving subsequent jump performance [23]. Rixon et al. [24] compared isometric vs. dynamic conditioning contractions and found an increase in CMJ height and peak power 3 min following three isometric MVC back squats; although 3 min after the 3RM dynamic back squats, there was no increase in CMJ height, and an increase in peak power was observed. However, the two conditioning activities were not identical in terms of volume to allow a direct comparison. Gourgoulis et al. [25] observed a significantly increased vertical jump performance following half squats with sub-maximal loads. In contrast, Hanson et al. [26] observed no significant increase in vertical jump performance following light (40%) and heavy (80%) load. Lower conditioning volumes may induce less fatigue and an earlier PAPE effect, although higher volumes may cause excessive fatigue and may delay the onset of PAPE or negate its presence [27,28]. The varied methodologies across studies, intensities and duration, as well as the equivocal findings in the literature, highlight the difficulty of comparing findings to determine an effective protocol to elicit PAPE.

Generating instability during a back squat exercise by suspending part of the total load from the barbell using elastic bands allows a higher activation of the stabilising muscles, as the lifter is likely to put greater effort into stabilising and controlling the bar [29]. The unstable load can negatively affect the range and speed of motion when compared to stable conditions to reduce force and power output [30]. However, Lawrence and Carlson [31] investigated the changes in force output and muscle activation during a back squat exercise at 60% of their 1-RM using unstable (i.e., elastic bands) and stable (i.e., free-weight) load and found a significant increase in muscle activity of the stabilising muscles (rectus abdominis, external obliques, and soleus). Therefore, the unstable load during the squat exercise incorporated as part of a warm-up can allow a greater activation of the stabilising muscles that may possibly contribute to subsequent performance enhancement.

The back squat exercise is commonly used to improve jump performance with Mina et al. [2] reporting that variable resistance (i.e., elastic bands attached equidistant to the sides of the bar and anchored to the floor) during a back squat exercise improved subsequent countermovement jump (CMJ) performance at 30 s, 4 min, 8 min, and 12 min compared to free-weight resistance alone. The increased muscle activation of vastus lateralis observed by Mina et al. [2] may have contributed to the increase in jump height, given the variation in muscle force requirements imposed by the use of variable resistance influenced the muscle recruitment patterns [2,9]. Therefore, the manipulation of different loading strategies during warm-up exercises may alter muscle recruitment amplitude, allowing increases in performance compared to traditional free-weight resistance alone [31,32].

It is of great importance for strength and conditioning practitioners to examine different variable resistance techniques as part of a warm-up routine to potentiate acute performance, enhance mechanical stimulus and muscle activity. However, no study to date has investigated the potential of suspending part of the total load from the barbell using elastic bands (i.e., unstable but constant load) in performance enhancement programmes. Therefore, the purpose of this study was to compare the influence of two back squat conditions; free-weight resistance (FWR) and unstable load (UN) suspended from the bar using elastic bands, following a comprehensive task-specific warm-up on subsequent CMJ performance. Given the improvements in performance previously reported after warm-up and conditioning contractions [1,2,31,32], it was hypothesised that (a) FWR and UN load would significantly improve subsequent CMJ performance (jump height, peak concentric power and RFD), and (b) the UN condition would provide significantly greater improvements than FWR condition.

## 2. Materials and Methods

### 2.1. Participants

Thirteen physically active men with more than two years’ resistance training experience (mean ± SD: age = 23.6 ± 1.6 years, height = 179.0 ± 9.2 cm, mass = 86.5 ± 10.0 kg) volunteered to take part in the current study. Inclusion criteria for participation were actively engaged with resistance training with experience in squat exercise and optimal training volume of 3–5 times per week but with no experience of using unstable load as part of their training program. The participants had to report no recent illness or lower limb injuries and refrained from engaging in strenuous activities and using stimulants for at least 48 h before the initial commencement of testing until completion of all testing sessions. Prior to the commencement of testing, all participants provided a written informed consent and completed a pre-medical questionnaire. Across all sessions, participants were instructed to wear the same footwear and were prohibited from using any supportive equipment. The study received ethical approval from the ethics committee at the University of Derby, United Kingdom, with approval reference ETH2122-0282.

To ensure an adequate population to reach statistical power (set at 0.8) was recruited, effect sizes were calculated for jump height (ES = 1.5), peak power (ES = 1.5) and RFD (ES = 1.3) using similar previous studies [2,33,34] with the measure with the smallest ES (i.e., RFD [ES = 1.3]) used to calculate sample size. The total sample size was estimated through a priori power analysis, using the G power V 3.1.9.7 software (Heinrich-Heine-Universität, Düsseldorf, Germany). The following input parameters were applied using a repeated-measure design: effect size f ≈ 1.34, α = 0.05, power = 0.80. The analysis revealed that the initial sample size required for statistical power was 10; therefore, considering the possibility of participant withdrawal and data loss, 15 participants were recruited with 13 participants completing the study.

### 2.2. Protocol Overview

To examine the acute effects of two different back squat conditions, control (FWR) or experimental (UN), a randomised crossover design was used on three separate occasions. Participants visited the laboratory for the familiarisation session and then either the FWR and UN conditions with a minimum separation of 72 h between each visit. Prior to all sessions, a comprehensive task-specific warm up was performed. During the familiarisation session, anthropometric data were collected, participants were familiarised with the testing protocols, and their one-repetition maximum (1-RM) back squat was assessed (please see below). In the experimental conditions, a prescribed warm-up routine was performed followed by three pre-intervention CMJs and then a set of three repetitions of back squat at 85% 1-RM (FWR and UN) followed by three post-intervention CMJs at 30 s, 4 min, 8 min, and 12 min.

### 2.3. Familiarisation Session and One Repetition Maximum (1-RM) Back Squat Assessment

During the familiarisation session, the participant’s 1-RM was assessed following a previously validated protocol designed by Sheppard and Tripplet [35]. Participants warmed up 5 min on a cycle ergometer (Monark 874E, Varberg, Sweden) at 65 rpm with a 1 kg load followed by 2 min rest and then performed two sets of 10 repetitions of unloaded back squat with a 20 kg Olympic bar with 2 min rest between sets. Participants then performed 8 to 10 repetitions at 50% of their previously determined 1-RM, and after a further 2 min rest, the load was increased by 10–20% for one set of 3 to 5 repetitions. Following a further 2 min rest, participants increased the load by 10–20% and performed one set of 2 to 3 repetitions. After 2–4 min rest, the load was increased by 10% and loads ~5% were added for each consecutive set of one repetition until failure to complete a lift. Their last successful lift was recorded as their 1-RM (144.23 ± 6.17 kg).

### 2.4. Comprehensive Warm-Up and Countermovement Jump Trials

In the FWR and UN conditions, a comprehensive task-specific warm-up was adopted from Mina et al. [2]. Participants performed a 5-min warm-up on a cycle ergometer at 60 rpm with a 1 kg load followed by 5 continuous body weight squats at 2:2 s tempo (eccentric/concentric). Following a 30 s rest period, participants performed another 5 continuous body weight squats at a 1:1 s tempo (eccentric/concentric). After a 20 s rest, 5 continuous CMJs at 70% of their perceived maximum were performed, and after a further 30 s rest, maximal CMJs were performed every 30 s until three consecutive jumps were performed within 3% of jump height. All participants completed 4–7 jumps in all trials. The CMJ was initiated from an upright position (keeping the hands on the hips at all times) and squatted downwards with the knees and hips flexed and jumped as high as possible, trying to reach maximal height [36]. To establish baseline (i.e., after warm-up) performance, data were collected 2 min later from three maximal pre-intervention CMJs. The procedures described above were followed by one of the conditioning contractions (described later), and a series of three maximum CMJ trials was performed at 30 s, 4 min, 8 min, and 12 min after the intervention with active recovery (i.e., walking) between each timepoint (see Table 1).

### 2.5. Intervention

**Figure 1:**
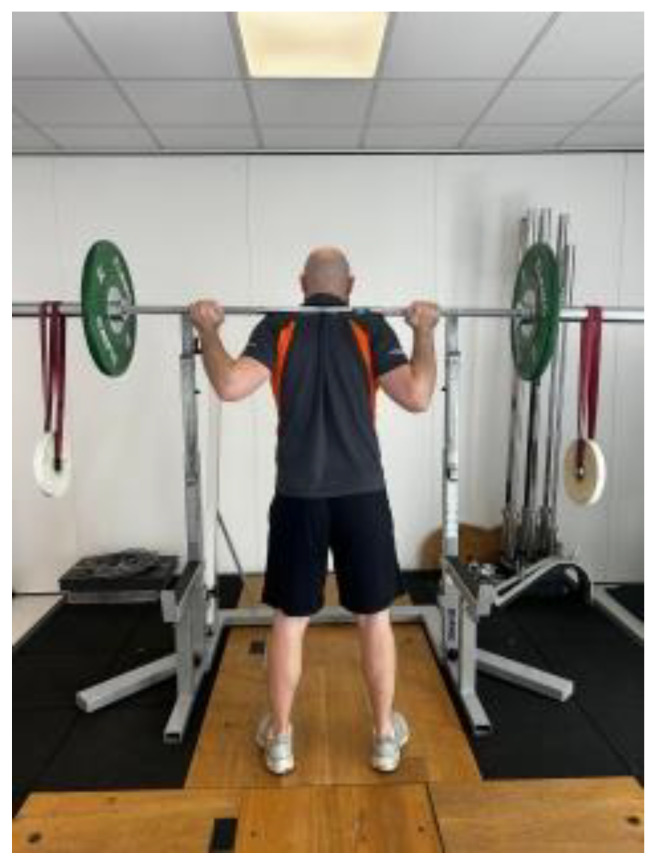
Unstable load from elastic bands hanging from the barbell during the back squat exercise.

In the FWR and UN conditions, participants performed one set of 3 repetitions of the back squat with the load set at 85% 1-RM. In the FWR condition, traditional load was added to the Olympic bar (20 kg) using weight plates set at 85% 1-RM ((0.85 × 1-RM load) − 20 kg) to determine the load on the bar. In the UN condition, the unstable load was set at 15% (0.15 × 0.85 × 1-RM load) and the remaining 85% load ((0.85 × 0.85 × 1-RM load) − 20 kg) was added using the traditional loading pattern (i.e., Olympic bar and weight plates). For example, where 1-RM is 100 kg load, in the FWR condition, this would equate to 85% 1-RM (85 kg) subtracting 20 kg (bar weight), leaving 65 kg on the bar. In the UN condition, the unstable load (15%) will require 13 kg of unstable load and the remaining 85% will be 72 kg, subtracting the 20 kg bar leaving 52 kg weight on the bar. The unstable load was suspended from the bar with the elastic bands placed next to the lifting collar with small diameter Eleiko plates hanging from the bar so that the load during the back squat exercise was not in contact with the floor. A super mini Pullum elastic band with ranging resistance of 10–50 lb resistance, 19 mm wide, 1041 mm long and with approximate distance from the bar at 60 cm on either side of the bar was used in this study (see Figure 1). Total loads in both experimental conditions were equal for each individual.

### 2.6. Force Platform Analyses

The kinetic data analyses were similar to Mina et al. [2]. During all CMJ trials, body mass was initially calculated with the participants standing stationary on the platform (Bertec, FP4060-10-2000, Bertec Corporation, Columbus, OH, USA) with ground reaction forces collected at a sampling frequency of 1000 Hz. Data processing initially included the participant’s weighting phase (i.e., body weight) [37], which was identified prior to the execution of each CMJ trial when the participants were stationary. The body weight was calculated by averaging the vertical ground reaction forces (GRFs) from each platform over a 2 s period and was divided by 9.81 to obtain each participant’s body mass. The net vertical force was calculated by subtracting the average body weight value from the vertical GRF value at each timepoint. Initiation of the jump (i.e., the beginning of the eccentric phase) was determined using the point when net vertical GRF decreased by two standard deviations (SD) below the mean baseline force (i.e., participant’s weight at rest) [2]. Vertical GRF was integrated during the eccentric and concentric phases of the jump using the trapezoid method. Impulse, which is equivalent to the change in momentum of the body, was then directly quantified by integrating the applied force over time using the following equation [38]:

J = ∫ Fdt = Δp,(1)

where J = impulse, F = force, t = time and ∆p = change in momentum.

Take-off velocity was then determined from impulse data by dividing by body mass, with jump height calculated from take-off velocity using standard equations for motion [39]. Since the force, mass, and initial velocity conditions were known, instantaneous velocity could be calculated. The instantaneous power was calculated as force × velocity, and the peak values were determined for the propulsive phase of the CMJ [2,38]:

V_(0)_ = 0, (2)

F_(i)_t = m(v_(i+1)_ − v_(i)_), (3)

Δv = (F_(i)_t)/m, (4)

P_(i)_ = F_(i)_ × V_(i)_, (5)

where F = force, t = 1/sampling frequency, m = mass of body, load, v = velocity, i = index value of the time series, and P = power.

The normalised (to body weight) peak RFD was calculated (eccentric and concentric phase) using a moving 20 ms time window from the first rise in force during the eccentric phase (2). The highest CMJ for each participant was identified at each of the five timepoints and the corresponding kinetic data were used for statistical analyses.

### 2.7. Statistical Analysis

The data obtained from the study were analysed using the SPSS statistical software (version 27.0; IBM, Armonk, NY, USA). All data are reported as mean ± standard error (SE) with eta squared (η_p_^2^) and Cohen’s d used to calculate effect sizes (ES) for the analysis of variance (ANOVA) and post hoc *t*-tests, respectively. Boundary intervals for η_p_^2^ effect sizes were <0.10 (negligible), 0.10–0.24 (small), 0.25–0.40 (medium), and ≥0.40 (large) for Cohen’s d boundary intervals were <0.2 (negligible), 0.2–0.49 (small), 0.5–0.79 (medium), and ≥0.8 (large) [40]. The Shapiro–Wilk test was used to assess normal distribution; no significant difference (*p* > 0.05) was observed in any variable indicating a normal distribution across all data sets. Mauchley’s tests were used to assess homogeneity of variance and where sphericity was violated, Greenhouse–Geisser (Epsilon ≤ 0.75) or Huynh–Feldt (Epsilon > 0.75) correction factors were used [40]. To determine differences in (a) jump height, (b) peak concentric power, and (c) RFD 20 ms, separate two-way repeated measures ANOVAs (time × condition) were performed. Where significant differences were detected, post hoc analyses with Bonferroni and Sidak corrections proved too conservative (i.e., masked the location of the difference); thus Tukey’s, LSD correction was used to determine the location of the differences. Statistical significance was set at *p* < 0.05 for all tests.

## 3. Results

### 3.1. Reliability

Within-session reliability for all measures was determined during pre-intervention (baseline) CMJ measures. No significant differences (*p* > 0.05) were detected in any data set with interclass correlation coefficients (ICCs) calculated for jump height (0.93), peak concentric power (0.89) and peak RFD (0.76) indicating good-to-excellent reliability with low coefficients of variance (CV) calculated for jump height (4.2%), peak concentric power (2.0%) and peak RFD (4.7%).

### 3.2. Jump Height

The two-way repeated measures ANOVA revealed no significant interaction effect (F_2.06, 24.74_ = 0.368, *p* = 0.702, η_p_^2^ = 0.030) for jump height, while a significant main effect of time (F_2.22, 26.63_ = 3.493, *p* = 0.041, η_p_^2^ = 0.225) but not of condition (F_1, 12_ = 1.873, *p* = 0.196, η_p_^2^ = 0.135) was detected. Post hoc pairwise comparisons revealed that Bonferroni and Sidak corrections were too conservative, as no significant difference at any timepoint was detected.

**Figure 2:**
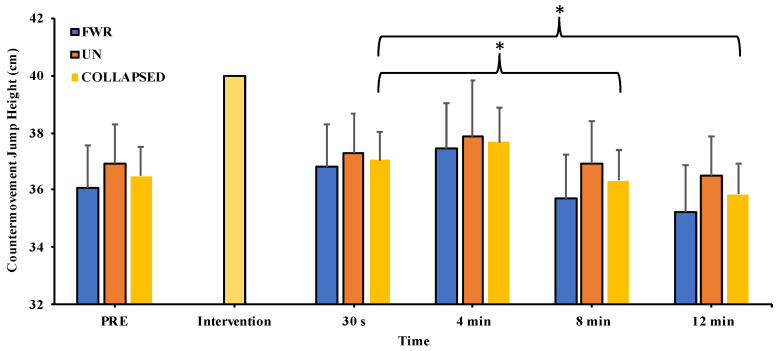
Measures of countermovement jump performance pre-intervention (PRE) and across all timepoints following the free weight resistance (FWR) and unstable (UN) conditioning interventions (collapsed data also shown). Values are presented as mean ± SE; * *p* < 0.05.

Tukey’s LSD revealed no significant difference at any timepoint (30 s, 4 min, 8 min, or 12 min) compared to pre-intervention (data collapsed across conditions: mean range = −1.7 to 3.2% (*d* = −0.35 to 0.36); FWR condition: mean range = −1.0 to 4.1% (*d* = −0.26 to 0.46); UN condition: mean range = −1.2 to 2.3% (*d* = 0.00 to 0.79)) (see Figure 2). However, pairwise comparisons revealed jump height was significantly higher at 30 s than at 8 min (data collapsed across conditions = 2.0 ± 1.2% (*d* = 0.35); FWR condition = 3.0 ± 2.0% (*d* = 0.48); UN condition = 1.0 ± 1.4% (*d* = 0.20)) and 12 min (data collapsed across conditions = 3.2 ± 1.2% (*d* = 0.58); FWR condition = 4.4 ± 2.0% (*d* = 0.68); UN condition = 2.1 ± 1.3% (*d* = 0.47)).

### 3.3. Peak Power

**Figure 3:**
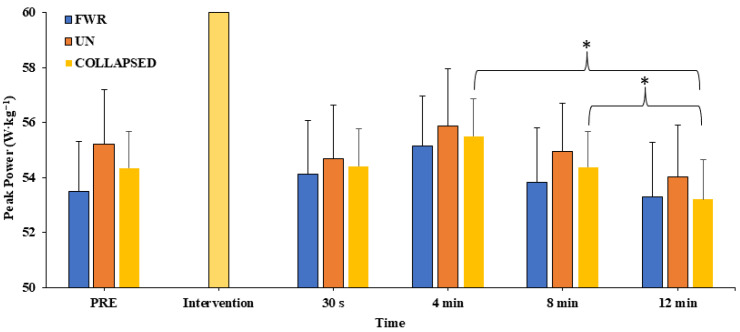
Measure of peak power pre-intervention (PRE) and across all timepoints following the free weight resistance (FWR) and unstable (UN) conditioning interventions (collapsed data also shown). Values are presented as mean ± SE; * *p* < 0.05.

No significant interaction effect (F_4, 48_ = 0.510, *p* = 0.729, η_p_^2^ = 0.041) was revealed for peak power, while a significant main effect of time (F_4, 48_ = 3.126, *p* = 0.023, η_p_^2^ = 0.207) but not of condition (F_1, 12_ = 3.400, *p* = 0.090, η_p_^2^ = 0.221) was detected. Post hoc pairwise comparisons revealed that Bonferroni and Sidak corrections were too conservative, as no significant difference at any timepoint was detected. Tukey’s LSD revealed no significant difference at any timepoint (30 s, 4 min, 8 min, or 12 min) compared to pre-intervention (data collapsed across conditions: mean range = −2.1 to 2.4% (*d* = −0.39 to 0.32); FWR condition: mean range = −0.3 to 3.6% (*d* = 0.06 to 0.36); UN condition: mean range = −4.0 to 1.3% (*d* = 0.12 to 0.74)) (see Figure 3). However, pairwise comparisons revealed peak power was significantly greater at 4 min (data collapsed across conditions = 4.2 ± 1.4% (*d* = 0.80); FWR condition = 3.3 ± 1.4% (*d* = 0.64); UN condition = 5.1 ± 1.4% (*d* = 0.94)) and 8 min (data collapsed across conditions = 2.3 ± 0.9% (*d* = 0.52); FWR condition = 0.9 ± 0.8% (*d* = 0.35); UN condition = 3.7 ± 1.6% (*d* = 0.67)) than at 12 min.

### 3.4. Peak RFD

**Figure 4:**
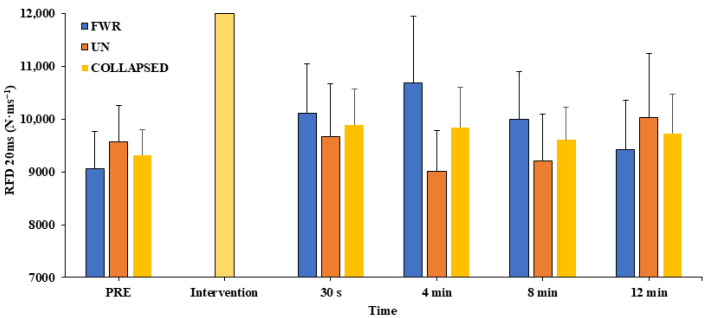
Measure of peak rate of force development (RFD) pre-intervention (PRE) and across all timepoints in the free weight resistance (FWR) and unstable (UN) conditioning interventions (collapsed data also shown). Values are presented as mean ± SE.

No significant interaction effect (F_4, 48_ = 1.447, *p* = 0.233, η_p_^2^ = 0.108) or main effects of time (F_4, 48_ = 0.294, *p* = 0.881, η_p_^2^ = 0.024) or condition (F_1, 4_ = 0.252, *p* = 0.625, η_p_^2^ = 0.021) were detected for peak RFD (see Figure 4).

## 4. Discussion

The current study investigated the magnitude and time-course of changes in countermovement jump (CMJ) performance after free-weight resistance (FWR) and unstable load (UN) back squat exercise performed following a comprehensive task-specific warm-up routine. In the FWR and UN conditions, no significant interaction or differences between conditions were detected at any timepoint. Using collapsed data (main effects analyses), compared to baseline, no significant changes were found in CMJ height, peak concentric power, or peak RFD at any timepoint (30 s, 4 min, 8 min, and 12 min), which is indicative of no potentiating effects of either intervention on CMJ performance. Thus, the hypotheses were rejected as neither the stable load during FWR or the unstable load during UN back squat exercise interventions enhanced CMJ performance. However, jump height at 12 min and 8 min was significantly lower compared to 30 s with peak power at 12 min also significantly lower compared to 4 min and 8 min. These reductions at 8 min and 12 min cannot be explained by fatigue, as no reduction was apparent at any earlier timepoint compared with baseline, and thus they are likely attributable to the participants losing motivation at 8 min and 12 min to perform numerous maximal CMJs at several timepoints. Regardless, the hypotheses were rejected, as neither the stable load during FWR or the unstable load during UN back squat exercise interventions enhanced CMJ performance. The lack of improvement compared to baseline in any measure following the FWR and UN condition after a comprehensive task-specific warm-up suggests that no additional benefit (i.e., PAP/PAPE effect) was derived from the inclusion of intense loading, which is consistent with previous research where an absence of change in CMJ performance was found when dynamic warm-up exercise (10 m lunge walks × 2, 10 body-squats × 2) was performed following high-intensity free weight contractions [41]. However, inconsistencies in PAPE responses [25,36,41] may depend on fatigue potentiation or perseveration potentiation interactions on subsequent performance.

Since this was the first PAPE study examining the impact of implementing unstable loads during the back squat exercise on jump performance, certain methodological approaches in this study could possibly explain the lack of significant changes following the UN condition. In the present study, the percentage of unstable loading was 15% of the 85% total load during the UN condition, which may be too low to sufficiently amplify potentiation and improve subsequent CMJ performance. Lawrence and Carlson [31] compared stable squats (traditional free-weights) and unstable squats (load suspended from the bar) with a load set at 60% of 1-RM and found an increased muscle activation of the torso (i.e., stabilising muscles), and during the pilot, >60% of 1-RM of five repetitions was perceived challenging for subjects to complete. Similarly, Ostrowski et al. [42] investigated stable and unstable bench press at two different intensities (60% and 80%) with greater muscle activation at 80% load in the concentric phase. This suggests that unstable loading techniques may vary across different exercises. However, previous research has extensively investigated variable resistance (i.e., chains and elastic bands) ranging 10–30% of the overall load [43,44]. Ebben and Jensen (2002) investigated elastic band and chain-loaded resistance set at 10% and found no significant effect on EMG or lifting kinetics [43]. Further, Stevenson et al. (2010) examined elastic band resistance set at 15% or 30% and failed to find a significant increase in power compared to FWR alone [44]. In contrast, which in combination with free-weights, they found no significant differences, although variable resistance set at higher percentages (35% load) has shown potentiating effects on squat performance [1,2,45,46]. In the present study, the amount of unstable load used was set at 15% of the total 85% given the challenging tolerance of using unstable loads alone at higher percentages [31,42], and the low proportion of unstable load failed to amplify potentiation.

Although the use of unstable load may require a greater muscle activation by the stabilising muscles, it can be more challenging for those who lack regular free-weight resistance training. In the present study, we used experienced weight-trained individuals, which could have allowed more control over the unstable load; hence, they may not have been unstable enough to elicit a potentiation response [47,48,49]. Therefore, the combination of unstable load with free-weight resistance can foster stability and reduce the degree of difficulty compared to unstable loads alone. Possible factors for this lack of difference could be the level of instability, intensity, elasticity of the bands suspending the load, movement tempo and the proportion of stable/unstable load. While power analysis was conducted, further study needs to be conducted with a larger sample size to confirm these data. Another limitation is that the participants had no previous experience with unstable load; thus, future investigations should allow a longer familiarisation period with unstable load. In addition, research is required to examine the electromyographic (EMG) activity of the stabilising muscles during stable and unstable load of the back squat exercise and how these may yield acute improvements in subsequent performance. In addition, considerations ought to include the type of conditioning contractions, including bench press, deadlift, etc. as well as participant characteristics (i.e., experienced versus novice lifters) to confirm the effectiveness of unstable load as a performance enhancement technique.

## 5. Conclusions

The use of FWR and UN load during the back squat exercise following a comprehensive task-specific warm-up failed to alter CMJ height and force/power production. These findings are suggestive that the proportion of unstable load used in the present study in combination with free-weights was insufficient to augment subsequent CMJ performance. Given the individuals that took part in the present study had over 2 years’ experience in weight training may have allowed a greater control over the unstable load; thus, the amount of unstable load may have been low to elicit a potentiation response. Further research is required to clearly understand how a higher proportion of unstable load in combination with free-weights during the back squat exercise can sufficiently challenge the musculature, the level of difficulty emanating from the unstable load, and the ability to maintain balance during the execution of the exercise to possibly increase subsequent performance.

## Figures and Tables

**Table 1:** Timeline of the study design.

Task	Intensity/Effort	Time [min]
5-min cycling	60 rpm	0–5.0
5 BW squats	2:2 s tempo	5.0–5.5
5 BW squats	1:1 s tempo	6.0–6.5
5 CMJs	70% perceived maximum	7.0–7.5
Single CMJs every 30 s	Maximum (100%)	8.0–8.5
CMJs (pre-intervention test)	Maximum (100%)	10.5–11.5
FWR or UN squats	85% 1-RM	12.5–13.0
CMJs (post-intervention test)	Maximum (100%)	13.5, 17.5, 21.5, 25.5

BW = body weight; CMJ = countermovement jump; FWR = free-weight resistance; UN = unstable load; 1-RM—one repetition maximum.

## Data Availability

Data are available upon request.
